# A Multi-Omics Prognostic Model Capturing Tumor Stemness and the Immune Microenvironment in Clear Cell Renal Cell Carcinoma

**DOI:** 10.3390/biomedicines12102171

**Published:** 2024-09-24

**Authors:** Beibei Xiong, Wenqiang Liu, Ying Liu, Tong Chen, Anqi Lin, Jiaao Song, Le Qu, Peng Luo, Aimin Jiang, Linhui Wang

**Affiliations:** 1 Department of Oncology, The First People’s Hospital of Shuangliu District, Chengdu 610200, China; xbb131@163.com; 2 Department of Urology, Changhai Hospital, Navel Medical University (Second Military Medical University), Shanghai 200433, China; 314007@163.com (W.L.); liuying03240@163.com (Y.L.); ct4899@foxmail.com (T.C.); song_jiaao@163.com (J.S.); 3 Department of Oncology, Zhujiang Hospital, Southern Medical University, Guangzhou 510280, China; smulinanqi0206@i.smu.edu.cn (A.L.); luopeng@smu.edu.cn (P.L.); 4 Department of Urology, Affiliated Jinling Hospital, Medical School of Nanjing University, Nanjing 210002, China; septsoul@smmu.edu.cn

**Keywords:** cancer stem-like cells, prognostic model, immune microenvironments, renal cell carcinoma

## Abstract

**Background:** Cancer stem-like cells (CSCs), a distinct subset recognized for their stem cell-like abilities, are intimately linked to the resistance to radiotherapy, metastatic behaviors, and self-renewal capacities in tumors. Despite their relevance, the definitive traits and importance of CSCs in the realm of oncology are still not fully comprehended, particularly in the context of clear cell renal cell carcinoma (ccRCC). A comprehensive understanding of these CSCs’ properties in relation to stemness, and their impact on the efficacy of treatment and resistance to medication, is of paramount importance. **Methods:** In a meticulous research effort, we have identified new molecular categories designated as CRCS1 and CRCS2 through the application of an unsupervised clustering algorithm. The analysis of these subtypes included a comprehensive examination of the tumor immune environment, patterns of metabolic activity, progression of the disease, and its response to immunotherapy. In addition, we have delved into understanding these subtypes’ distinctive clinical presentations, the landscape of their genomic alterations, and the likelihood of their response to various pharmacological interventions. Proceeding from these insights, prognostic models were developed that could potentially forecast the outcomes for patients with ccRCC, as well as inform strategies for the surveillance of recurrence after treatment and the handling of drug-resistant scenarios. **Results:** Compared with CRCS1, CRCS2 patients had a lower clinical stage/grading and a better prognosis. The CRCS2 subtype was in a hypoxic state and was characterized by suppression and exclusion of immune function, which was sensitive to gefitinib, erlotinib, and saracatinib. The constructed prognostic risk model performed well in both training and validation cohorts, helping to identify patients who may benefit from specific treatments or who are at risk of recurrence and drug resistance. A novel therapeutic target, SAA2, regulating neutrophil and fibroblast infiltration, and, thus promoting ccRCC progression, was identified. **Conclusions:** Our findings highlight the key role of CSCs in shaping the ccRCC tumor microenvironment, crucial for therapy research and clinical guidance. Recognizing tumor stemness helps to predict treatment efficacy, recurrence, and drug resistance, informing treatment strategies and enhancing ccRCC patient outcomes.

## 1. Introduction

Renal cell carcinoma (RCC), categorized as one of the top 10 common neoplasms worldwide, is projected to gain 79,000 fresh diagnoses in 2022 [1,2]. Approximately 75% of these incidents arise from clear cell RCC, which is the prevalent histological variant [1]. The tendency of ccRCC to metastasize and the consequent poor prognosis post-metastasis is a cause for concern. Close to one-third of the patients show signs of metastasis during the inaugural visit, with a similar portion experiencing post-surgical recurrence and metastasis [3,4]. Regrettably, the survival rate of advanced cases hovers around a mere 10% over five years [1,5]. ccRCC exhibits a pernicious insensitivity to standard chemoradiotherapy [6]. Therefore, the current leading-line therapeutic method favors the combination of immune checkpoint inhibitors and tyrosine kinase inhibitors. While this treatment has significantly improved patient outcomes, some patients may experience inherent or developed drug resistance following therapy [7,8,9]. Identifying such patients on time can enhance prognosis plans. Currently, the Fuhrman nuclear grading system and TNM grading system serve as the standard methods for risk stratification and prognosis prediction in clinical applications. However, these techniques overlook intra-tumoral molecular heterogeneity, leading to patients with similar clinical indicators to experience largely varied prognoses [10,11]. Therefore, there is an urgent need to find molecular markers related to prognosis and to elucidate related molecular mechanisms, so as to provide new diagnosis and treatment options for ccRCC.

CSCs embody a concept first introduced by Mackillop in 1983. This concept postulates that a unique subset within tumor tissues possesses stem cell properties. These properties are intricately associated with the resistance of tumors to both chemotherapy and radiotherapy, as well as with their metastatic capability and their inherent potential for self-renewal and differentiation [12]. The theory regarding the origin of CSCs suggests a link to epithelial–mesenchymal transition (EMT). This biological process holds crucial importance for the preservation of tumor cell stemness [13]. Additionally, developmental signaling pathways, such as Notch, WNT, Hedgehog (HH), and Hippo, are key players in the governance of the stem-like qualities of tumor cells [14]. The crosstalk between *NF-κB*, *MAPK*, *PI3K*, and *EGFR*-level pathways and other pathways also exerts influence over these stem-like characteristics [13,15,16]. Interactions between CSCs and the immune system are evident as well. For instance, tumor-associated macrophages (TAMs) have been observed to bolster proliferation in CSCs derived from hepatocellular carcinoma via IL-6 signaling [17]. Furthermore, dendritic cells (DCs) are implicated in fostering chemoresistance and tumorigenic properties in follicular lymphoma CSCs [18]. Moreover, regulatory T cells (Tregs) appear to augment colorectal cancer CSC functionalities by discharging IL-17 [19]. Several clinical trials are actively exploring inhibitors targeting CSCs in tandem with immune agents [NCT03548571, NCT02541370, NCT03739606, and NCT03386513]. Nonetheless, the mechanisms underlying CSC development and progression in renal cell carcinoma remain to be fully elucidated. Challenges are compounded by the scant presence of CSCs, rendering research at the tissue level more complex. The attributes of CSCs may, however, be effectively analyzed by examining their surface markers and employing less labor-intensive techniques.

In our investigation, we segregated patients afflicted with ccRCC via the amalgamation of comprehensive omics evidence, such as molecular attributes, cellular biological roles, immunity incursion, and sensitivity testing to pharmacological treatments. This approach was designed to delve into the cellular–molecular processes underpinning stemness traits in tumors, stratified across various cohorts. Additionally, we formulated a robust prognostic model to corroborate the pivotal function of stemness indicators in foretelling the clinical outcomes for individuals with ccRCC. Beyond its prognostic utility, this construct is also instrumental in predicting potential therapeutic ineffectiveness attributable to the emergence of drug resistance.

## 2. Method and Materials

### 2.1. Data Collection and Processing

In conducting this research, multiple established databases were harnessed to gather data pertaining to ccRCC. We sourced normalized gene expression patterns, epigenetic DNA methylation profiles, as well as tumor mutational burden (TMB) and relevant clinical information, specifically for the ccRCC patient group, from the UCSC Xena platform [20]. Data on copy number variations (CNV) and genetic mutations were also extracted from the Genomic Data Commons (GDC) database with the use of the R package TCGAbiolinks. For insights into the Japanese renal cancer population, we obtained gene expression and clinical data available under the accession number PHS002252.V1.P1 [21]. Single-cell RNA sequencing data specific to ccRCC were collected from the GEO repository, with the entry number PRJNA705464 [22]. The analysis was further enriched by integrating data from a range of public platforms, including UALCAN, TIMER, TIDE, and MEXPRESS. It is pertinent to acknowledge that these datasets, being publicly available, do not require informed consent or Institutional Review Board (IRB) approval for use. The StemChecker webserver (http://stemchecker.sysbiolab.eu, accessed on 10 February 2024) compiled 26 stemness signatures (*Homo sapiens*) derived from various methodologies, which include expression profiles, computational derivation, literature curation, transcription factor target genes, and RNAi screen procedures.

### 2.2. Identification of Different Subsets in ccRCC

To investigate the relationships among stemness-associated signatures in ccRCC, we calculated the stemness score for each sample using gene set variation analysis. We then utilized a stemness-related matrix to identify heterogeneous subtypes of ccRCC through consensus clustering, as implemented in the ConsensusClusterPlus package with the following parameters: maxK = 10, reps = 1000 bootstraps, pItem = 0.8, pFeature = 1, clusterAlg = “km”, and distance = “euclidean”. The criteria for determining clustering features included: (1) a significant distinction between tumor and normal samples and (2) the preservation of heterogeneity across all tumor samples. The clustering process generated co-classification matrices for various cluster sizes (K ranging from 2 to 10). The algorithm executed 1000 iterations with a resampling rate of 0.8, employing the partitioning around medoids method along with a 1-Spearman correlation distance. The optimal number of clusters was selected based on the consensus score matrix and the cumulative distribution function (CDF) curve, facilitating a robust classification of ccRCC subtypes.

### 2.3. Enrichment Analysis between Subgroups

For the purpose of pinpointing genes expressed differentially between the categorized subgroups, we leveraged the R-based “DESeq2” package. We set forth rigorous selection standards, specifically an adjusted *p*-value cut-off below 0.01, coupled with a requirement for the fold-change to exceed a value of 2. Subsequent to identifying these DEGs, we put to use the “ClusterProfiler” package within R to carry out detailed functional enrichment inquiry. Our procedures included analyses, such as gene ontology (GO), Kyoto Encyclopedia of Genes and Genomes (KEGG) pathway analysis, gene set enrichment analysis (GSEA), and gene set variation analysis (GSVA), with the intent of unearthing the biological activities and pathways responsible for the disparities between the subgroups CRCS1 and CRCS2. The GMT files requisite for these enrichment endeavors were retrieved from the Molecular Signatures Database and ConsensusPathDB [23].

### 2.4. Differences in Characteristics of Immune Infiltration and Response to Treatment

To evaluate the intricacies of the immune landscape and discern cellular infiltration in clear cell renal cell carcinoma (ccRCC) tissue samples, a battery of analytical tools was deployed. This array included computational models, like TIMER, CIBERSORT, QUANTISEQ, MCPCOUNTER, XCELL, and EPIC, all designed to measure the presence and proportion of immune cells, yielding enrichment scores that facilitate a side-by-side assessment of the tumor microenvironment in the distinct subgroup populations. Furthermore, we conducted single-sample gene set enrichment analysis (ssGSVA) to validate the differential immune infiltration distinguishing the subgroups CRCS1 and CRCS2. In an effort to deepen our understanding of the immune milieu, the “ESTIMATE” package in R was used to extract both stromal and immune scores from the gene expression data of ccRCC tissues. These scores serve as indicators of the non-tumor cell constituents within the cancer microenvironment. We then referred to the Tumor Immune Dysfunction and Exclusion (TIDE) resource, accessible at http://tide.dfci.harvard.edu/ (accessed on 20 February 2024), to appraise the likelihood of distinct immune-therapeutic outcomes among the cohorts being examined [24]. Employing this multifaceted methodology affords a comprehensive view of the varied elements at play within the tumor microenvironment and how they might influence the effectiveness of applied treatments.

### 2.5. Characteristics of Mutation Spectrum among Subpopulations

Our investigation delved into the mutational spectra of the study’s designated subgroups, with the “Maftools” package in the R programming language serving as our primary tool for mutational data visualization and analysis [25]. The DepthOfCoverage function in the Genome Analysis Toolkit (GATK v3.8.1.0) was utilized for computing read-depth statistics. Paired-end reads, in the Fastq format, were mapped to a human reference genome (UCSC Genome Browser, hg38) employing the Burrows–Wheeler aligner. Following GATK’s recommended best practices, variant calling was implemented. MuTect2 (GATK v4.1.2.0) was employed for the identification of somatic single-nucleotide variations and minor insertions and deletions, which were then annotated with ANNOVAR using known genes from UCSC as references. The R package Maftools (v3.10) was used to visualize mutant genes possessing non-synonymous mutations. MutSigCV80 was applied for determining significantly mutated genes utilizing standard parameters, identifying genes with a Benjamini–Hochberg-adjusted *p*-value less than 0.01 as significantly mutated ones. Via the somaticInteractions function in the Maftools R package and using Fisher’s exact test for pair-wise comparisons, sets of genes that are mutually exclusive or which co-occur were discovered. A threshold of *p* < 0.05 determined statistical significance. Additionally, we computed survival rates associated with gene mutations, delving into the potential prognostic significance of particular mutational signatures referenced in existing scientific discussions. The “Maftools” package’s functionality was also extended to examine how genetic aberrations correlate with drug response, and to contrast oncogenic pathway activities between subgroups. For a sophisticated examination of genomic structural changes, we implemented the GISTIC 2.0 algorithm, which allowed for the measurement of Euclidean distances centered on well-defined threshold copy number variations. Pursuant to this method, we were able to pinpoint recurring and focal copy number alterations (SCNA) among our study cohorts [26].

### 2.6. Processing of Spatial Transcriptomics Data

Drawing on existing studies and spatial transcriptomic data from RCC models, we executed an exhaustive examination to uncover the intricate molecular configuration [27]. For this purpose, the Seurat (v4.1.0) package in R was employed to normalize the spatial transcriptomics datasets via logarithmic transformation, ensuring both consistency and ease of comparison. Following this, we amalgamated Seurat objects into a consolidated spatial transcript (ST) compilation, using the SelectIntegrationFeatures, FindIntegrationAnchors, and IntegrateData functions. These actions were aimed at synchronizing the data across disparate batches and minimizing batch-related discrepancies. To simplify the data’s complexity, dimensionality reduction was performed using RunPCA. Afterward, by leveraging the FindNeighbors and FindClusters functions, we classified ST points into various clusters based on their distinct molecular signatures. The initial segregation of clusters was informed by a histological review of hematoxylin–eosin (H&E) stained specimens, with further refinement achieved via unsupervised cluster analysis. Throughout the process of assigning cellular markers to clusters, we observed clusters with pronounced expressions of numerous cell markers, hinting at cellular diversity. To tackle this complexity, the ssGSEA algorithm was utilized. This method facilitates the computation of scores for prevalent cell types by averaging the expression patterns seen across different clusters. This strategy emerged as notably effective and insightful within the realm of spatial transcriptomics, offering a detailed portrayal of the cellular array present within each cluster [28].

### 2.7. Drug Susceptibility Analysis

To facilitate the subtype-based targeted interventions, we introduced an integrated pipeline to identify potential therapeutic agents for each subtype of ccRCC. We applied the R package pRRophetic to predict patients’ response to preclinical and clinical chemotherapeutic agents from the Cancer Drug Sensitivity Genomics (GDSC) database based on the tumor gene expression profiles of CRCS1 and CRCS1. In detail, the model used for predicting drug response was ridge regression model implemented in the pRRophetic package [29]. This predictive model was trained on mRNA expression profiles and drug response data of cancer cell lines with a satisfied predictive accuracy, which were evaluated by default 10-fold cross-validation, thus allowing the estimation of clinical drug response using only patients’ baseline gene expression data. To explore the connections between gene expression levels and drug reactivity, we turned to the Spearman correlation coefficient. Through this statistical tool, we were able to discern whether there was a link between the heightened expression of certain genes and drug resistance or susceptibility. Specifically, a positive Spearman correlation was indicative of greater gene expression aligning with a resistance to the drugs in question, while a negative correlation pointed towards a heightened sensitivity to the treatments under consideration.

### 2.8. Construction of Risk Prediction Model

To identify potential biomarkers for predicting patient survival times in cases of renal cell carcinoma, our research began with a univariate Cox regression analysis, focusing on markers relevant to distinct subgroups within the TCGA-KIRC dataset. The goal was to pinpoint variables with a significant correlation to patient survival rates. Specifically, the construction of our risk prediction model was conducted in two phases. Initially, we employed the R package ‘survival’ to extract prognostically relevant features (Cox.*p* value cutoff = 0.05). Subsequently, we utilized the R package ‘randomForestSRC’ to further reduce the number of features (Ntree = 1000). In the subsequent step, the random survival forest variable hunting (RSFVH) approach—an advanced machine learning algorithm—was applied to further distill and confirm the most pivotal prognostic indicators drawn from the data. Leveraging these findings, we crafted a detailed risk prediction model that consolidates these crucial prognostic genes into a singular, effective prognostication tool. We conducted rigorous validation of this model utilizing an independent dataset, sourced from the JAPAN-ccRCC study, thereby affirming its reliability and transferability across varied patient demographics. To translate the model’s prognostic output into practical clinical applications, patients within both the TCGA-KIRC and JAPAN-ccRCC groups were divided into higher and lower risk categories. This division was based on the median of the risk score calculations. Through such stratification, we are able to support more customized patient care and enrich our comprehension of renal cell carcinoma’s prognostic factors.

### 2.9. Statistical Analysis

Statistical analyses, data processing, and visualizations were performed using R version 4.0.4. We assessed correlations between continuous variables with Spearman’s correlation coefficient. Normality tests were conducted on the datasets prior to analysis. For data conforming to a normal distribution with homogeneity of variances, comparisons between two groups were made using the Student’s *t*-test, while one-way ANOVA was employed for multiple group comparisons. In cases where the data did not meet these assumptions, the Wilcoxon rank-sum test was used for two-group comparisons, and the Kruskal–Wallis test was utilized for multiple groups. Categorical variables were evaluated using either the Chi-square test or Fisher’s exact test, depending on the data structure. Furthermore, for patient survival analysis—encompassing both overall survival (OS) and progression-free survival (PFS)—we adopted the Kaplan–Meier estimator. Derived *p* values were adjusted for false discovery rates (FDR) using the Benjamini–Hochberg method, and an FDR threshold of 0.1 was set for significance, as our previous works reported [30,31,32]. All codes relevant to this work were deposited in https://github.com/jaingaimin/ccRCC_stemness (accessed on 10 September 2024).

## 3. Results

### 3.1. Two Clusters of ccRCC Were Identified by Clustering Analysis of Tumor Stemness Signatures

**Figure 1:**
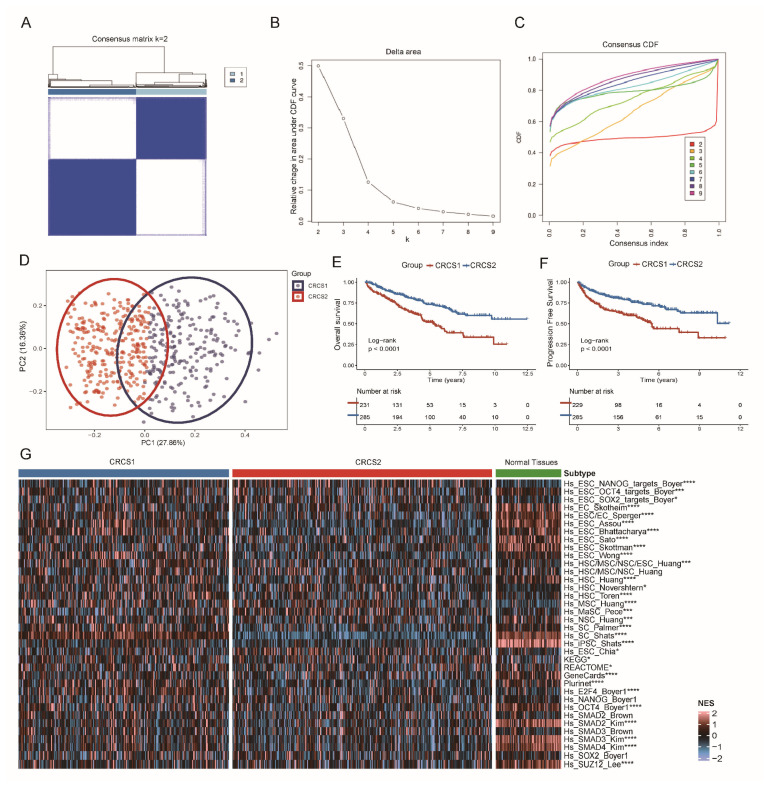
Identification of novel stemness-related subgroups among clear cell renal cell carcinoma patients. (**A**) The consensus score matrix based on stemness-related signatures when k reaches 2. (**B**) The relative change in the area under the CDF curve from 2 to 9. (**C**) The cumulative distribution function (CDF) plots for varying k values, each delineated with a unique color, contribute to cluster robustness evaluation and assist in pinpointing the optimal k value. (**D**) A two-dimensional principal component analysis (PCA) plot based on stemness scores distinguishes the subtypes. (**E**,**F**) TCGA-KIRC cohorts’ Kaplan–Meier curves for OS and PFS. (**G**) A heatmap presents a comparative display of stemness signals across CRCS1, CRCS2, and normal tissue counterparts. * *p* < 0.05, *** *p* < 0.001, **** *p* < 0.0001.

Samples of TCGA-ccRCC were stratified into various molecular classifications based on tumor stemness, employing a method of unsupervised clustering. Figure 1A–D illustrates the division of ccRCC into two principal clusters, specifically named tumor stemness-related cancer subtype 1 (CRCS1) and CRCS2. We evaluated the clinical impact of this subtyping by examining the outcomes between these two dominant classifications (Figure 1E,F). Our findings showed that the CRCS2 subgroup demonstrated lower T stages and grades in contrast to CRCS1 (Table 1), offering a conspicuously superior survival benefit. Moreover, we scrutinized the gene expression related to tumor stemness among these two subtypes of ccRCC and within standard tissue samples. Subtype CRCS2 was characterized as having markedly reduced stemness attributes, displaying a scarcity in gene expression levels when juxtaposed with CRCS1 and normal tissue. Significantly, Hs_SC_Palmer gene expression was found to be substantially heightened in normal tissue as well as in patients from the CRCS1 group, compared to those categorized under CRCS2. On the other hand, the Hs_ESC_NANOG_targets_Boyer gene was notably more active in patients from the CRCS2 group, insinuating that an atypical level of tumor stemness could be linked with improved prognoses (Figure 1G).

### 3.2. Functional Enrichment Analysis of Different Tumor Stemness Subgroups

**Figure 2:**
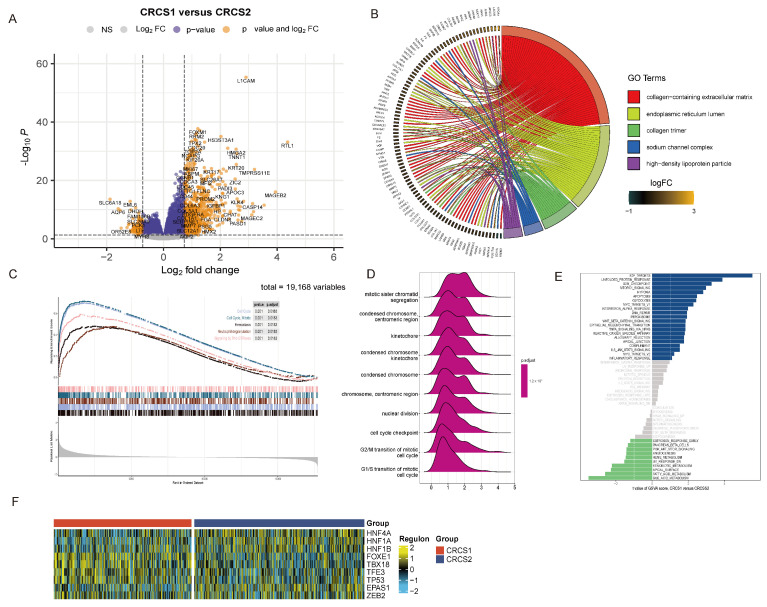
Functional enrichment analysis. (**A**) Volcano plot shows different expression genes between CRCS1 and CRCS2. (**B**) Functional enrichment of gene ontology and gene set enrichment analysis (**C**,**D**), and gene set variation analysis (**D**) of DEG. (**E**) GSVA analysis of DEG between CRCS1 and CRCS2. (**F**) The heatmap summarizes the differing regulation of transcriptome factors between CRCS1 and CRCS2.

Considering the distinct clinical features of each subgroup, we sought to define the disparate gene expression profiles for CRCS1 and CRCS2. We collated genes exhibiting aberrant expression for a subsequent functional enrichment assessment (Figure 2A). The GO enrichment analysis disclosed that the genes in question participate in a spectrum of pathways, including the collagen-containing extracellular matrix and the endoplasmic reticulum lumen at the cellular component level. Biological processes, such as pattern specification and extracellular matrix organization, were highlighted alongside molecular functions, like glycosaminoglycan binding and peptidase inhibition activities (Figure 2B and Figure S1A,B), predominantly implicating cellular cycle processes. GSEA was performed to enrich pathways based on differential gene expression, revealing that CRCS1 was significantly associated with cell cycle progression, mitosis, coagulation, neutrophil activation, and Rho GTPase signaling (Figure 2C). Concurrently, KEGG analysis confirmed ties to cell division processes, like sister chromatid segregation, the condensed chromosome centromeric region, and kinetochore-related pathways (Figure 2D). By applying GSVA, we assessed the disparity between gene sets, discerning that pathways, such as E2F Targets, unfolded protein response, and G2M checkpoint control, were prominently heightened in CRCS1. In contrast, metabolic pathways, like bile acid and fatty acid metabolism, as well as the apical surface pathway, were significantly more active in CRCS2 (Figure 2E). To probe the divergences at the transcriptomic level, regulon analysis of transcription factor gene sets was conducted. This revealed that factors, such as HNF4A, HNF1A, HNF1B, and EPAS1, were elevated in CRCS2, while FOXE1, TBX18, TFE3, and TP53 were more active in CRCS1 (Figure 2F). EPAS1, known as a regulator of genes under hypoxic stress, sees an increase in expression as oxygen levels diminish [33], signaling a hypoxic environment in CRCS2. Tumor hypoxia is recognized for driving resistance to immunotherapies [34,35], suggesting that tempering hypoxia might prime CRCS2 for better immunotherapy responsiveness. Moreover, the HNF1A gene is linked to glucose metabolism [36] while TFE3 has a strong association with Xp11 translocation renal carcinoma, characterized by TFE3 gene fusion [37].

### 3.3. Comparison of Specific Immune Infiltration in Two Subgroups

**Figure 3:**
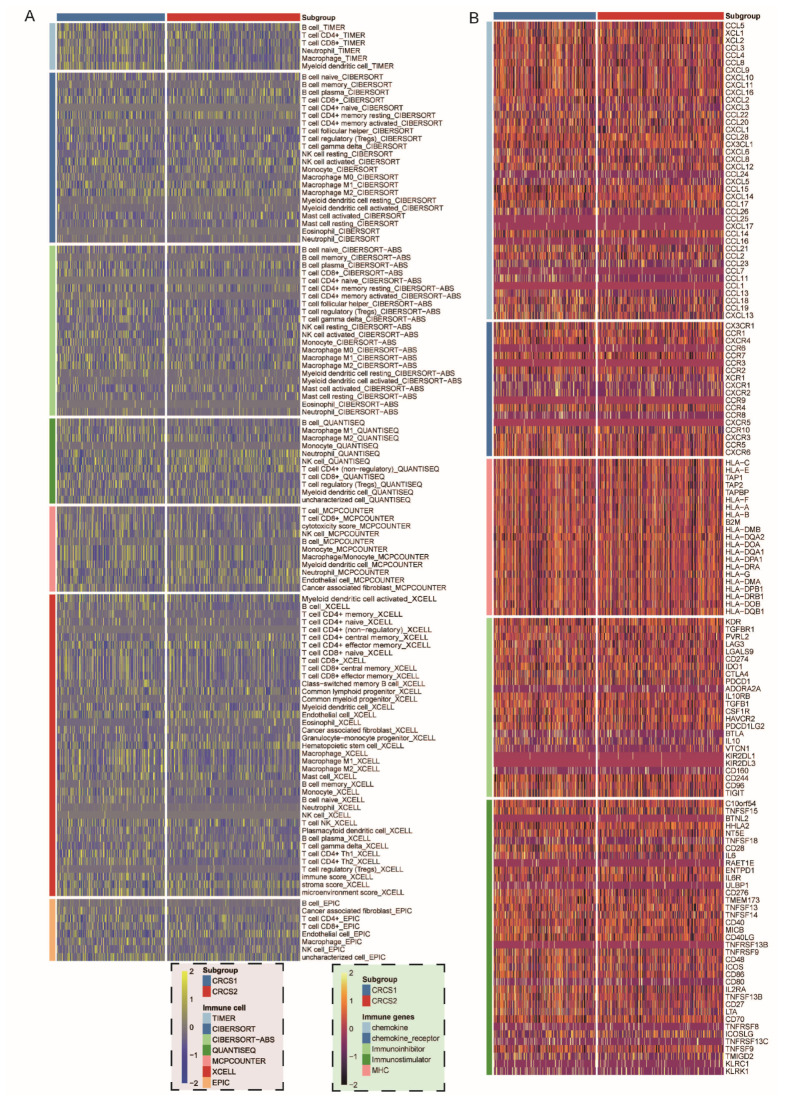
Immune landscape of CRCS subtypes. (**A**) Heatmap of relative immune infiltration and expression levels of immune-related genes (**B**) between CRCS1 and CRCS2.

**Figure 4:**
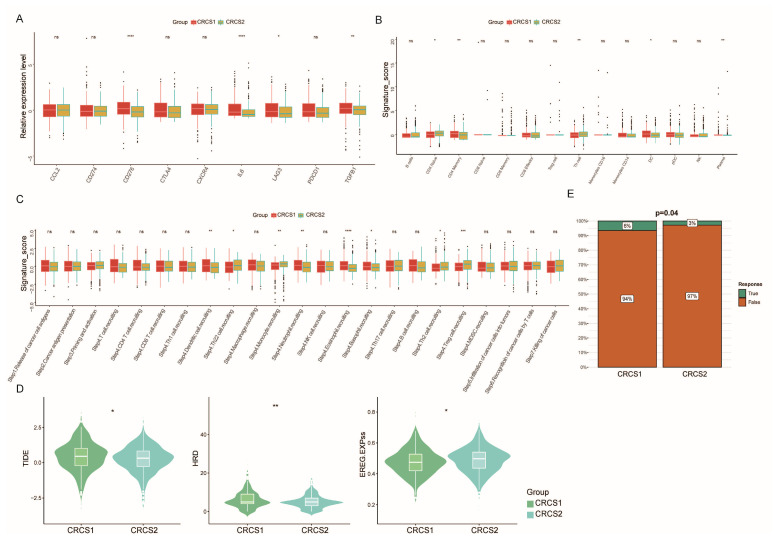
Different immune landscapes between groups. (**A**) Boxplot of the immune checkpoint-related genes and immune cell infiltration degree (**B**) and anti-tumor related cancer immune circulation (**C**) between CRCS1 and CRCS2. (**D**) Boxplot of TIDE, HRD, and EREG stemness scores between CRCS1 and CRCS2. (**E**) TIDE analysis suggested that the CRCS1 could be more sensitive to ICI therapy. ^ns^ *p* > 0.05, * *p* < 0.05, ** *p* < 0.01, *** *p* < 0.001, **** *p* < 0.0001.

A suite of algorithms was applied to dissect the immune infiltration profile within the two cohorts, focusing on characterizing the variety of cellular players within the tumor microenvironment (TME). The analysis conveyed a clear pattern showing that CRCS2 exhibited a diminished presence of B cells, T cells, monocytes, and a range of other immune cells compared to CRCS1 (Figure 3A,B). Concurrently, the levels of immune markers, such as CD276, IL6, and TGFB1, were found to be appreciably lower in CRCS2 relative to CRCS1 (Figure 4A). The CRCS1 subgroup was marked by an increased abundance of memory CD4^+^ T cells, posing an implication for its potential receptivity to immunotherapy. Coupling these insights with survival and functional analysis led to the postulation that CRCS2 might represent an immune desert phenotype, defined by its immunosuppressed state. In further evaluating cell recruitment activity, we noted that CRCS2 had reduced expression of genes related to eosinophil and neutrophil recruitment. In contrast, the recruitment genes for regulatory T (Treg) cells and monocytes were more upregulated in CRCS2 than in CRCS1 (Figure 4C). Moreover, the CRCS1 subgroup exhibited higher scores in the Tumor Immune Dysfunction and Exclusion (TIDE) and ENHss analyses, while CRCS2 scored higher in EREG.EXPss and HRD (Figure 4D). These findings suggest that CRCS1 is characterized by a simultaneous state of elevated immune infiltration and immunological dysregulation. Analysis of immunotherapy response rates further highlighted the differences between the subtypes, with CRCS1 showing significantly higher rates than CRCS2 (6% versus 3%, respectively, as shown in Figure 4E). Therefore, these findings underscore the notion that disparate immune landscapes correlate with variations in tumor stemness profiles.

### 3.4. Characterization of Somatic Mutations and CNV in Two Clusters

**Figure 5:**
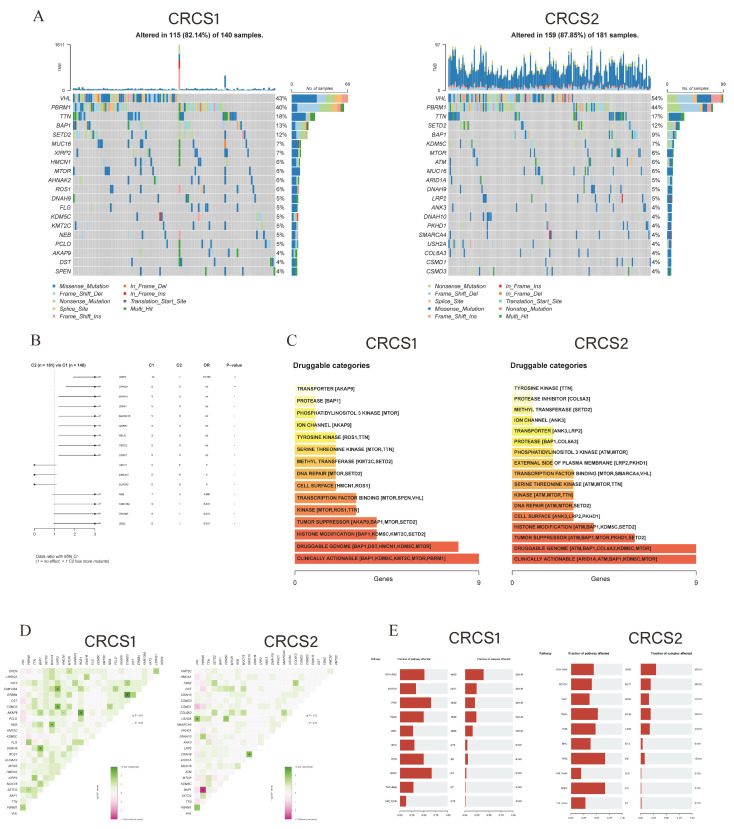
The genomics alteration between different groups. (**A**) The waterfall plot shows the different mutation landscapes of the top 20 most recurrently mutated genes in CRCS1 and CRCS2. (**B**) The forest plot illustrates the prognostic relevance of key mutated genes, comparing their effects on survival between the subtypes. * *p* < 0.05, ** *p* < 0.01. (**C**) Analysis of the mutation data in both CRCS1 and CRCS2 subtypes leads to the identification of potential druggable gene targets, categorized by their likelihood of being actionable. (**D**) Somatic mutations’ prevalence and patterns, including co-occurring and mutually exclusive events, are presented in a heatmap for the top 25 frequently mutated genes. (**E**) Pivotal oncogenic pathways’ mutations in CRCS1 and CRCS2.

**Figure 6:**
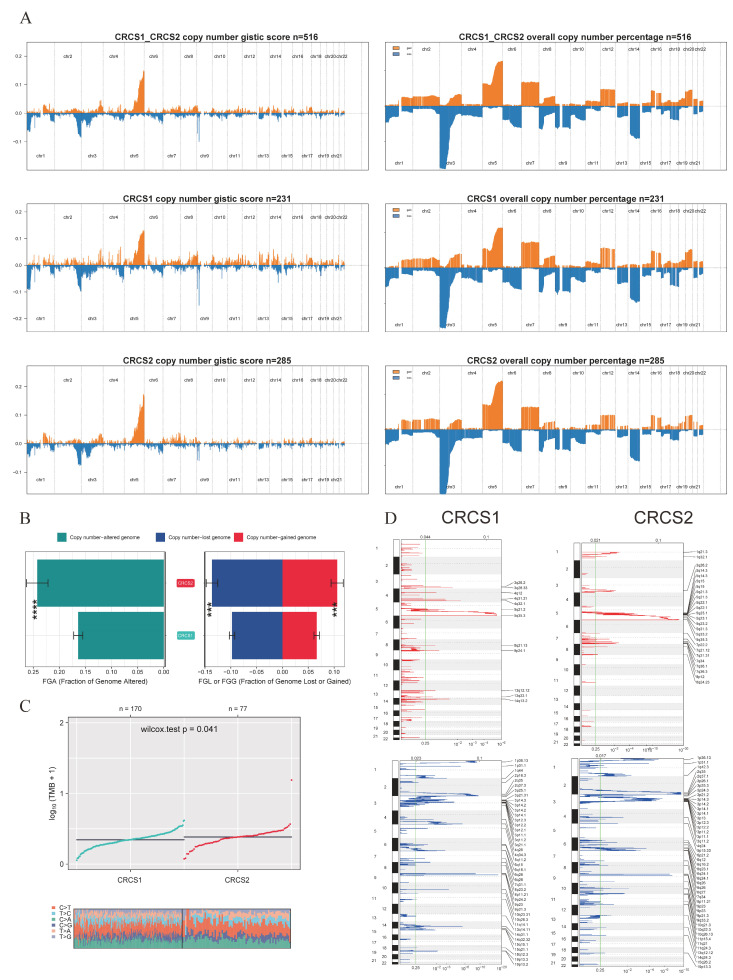
Copy number variation between different groups. (**A**) Detailed CNV and total CNV (**B**) frequency between CRCS1 and CRCS2. (**C**) Different tumor mutation burden within each subtype. (**D**) CNV frequency at each genome regions between CRCS1 and CRCS2 (red represents increased copy number, while blue represents decreased copy number). *** *p* < 0.001, **** *p* < 0.0001.

An in-depth analysis was conducted to unravel the distribution of somatic mutation disparities between the two focal groups. The comprehensive survey unveiled the 20 most commonly mutated genes, showcasing that CRCS1 exhibited a lower overall mutation frequency than CRCS2 (Figure 5A). Notably, CRCS2 displayed higher prevalence rates for specific mutated genes, like VHL, PBRM1, and ATM, as opposed to CRCS1 (Figure 5B). Delving into potential therapeutic targets based on mutational data, we harnessed the DGIdb database and maftools package for investigating drug interactions. The relatable gene sets for therapeutic intervention were classified into 15 classes for CRCS1 and 17 classes for CRCS2, encompassing clinically actionable targets, tumor suppressors, histone modification elements, and DNA repair candidates, among others (Figure 5C). Somatic interaction exploration exposed co-mutations, such as those involving PBRM1 and BAP1, as well as VHL and CSMD1, known to prompt cell demise in CRCS2, delineating a potential therapeutic avenue for this subset (Figure 5D). Employing the maftools R software package further illuminated the somatic alterations in rare oncogenic pathways within both groups, spanning pathways, like RTK-RAS, NOTCH, PI3K, Hippo, WNT, MYC, TP53, NRF2, TGF-Beta, and Cell Cycle. Noteworthy changes were observed in NRF2 and PI3K for CRCS1, whereas TP53 and NRF2 emerged as the most notably altered oncogenic pathways in CRCS2 (Figure 5E). Profiling the copy number variant (CNV) distinctions between the clusters showcased a heightened CNV rate in CRCS2 (Figure 6A). Decoding the amplification and deletion zones on chromosomes for each group utilizing GISTIC 2.0 displayed congruous patterns in percentage gain/loss and GISTIC scores (Figure 6A–D). These discernments suggest that dissimilar CNV occurrences potentially contribute to the distinctive formations observed in both isoforms.

### 3.5. Drug Susceptibility Profiles of Different Stemness Subsets

**Figure 7:**
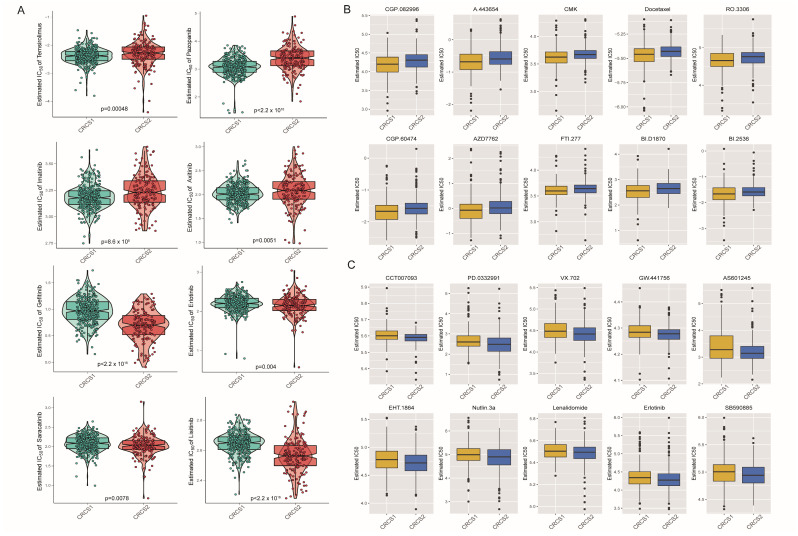
Evaluating the therapeutic agents’ benefit for each group. (**A**) Violin-box plot indicates different sensitivity to agents which introduced in clinical practice. (**B**) Potential pre-clinical agents which are sensitive to CRCS1 and CRCS2 (**C**) from the GDSC database.

In the drug sensitivity assessment, we utilized drug response specifics, delineated by IC50 values, garnered from the GDSC database. Analysis pointed towards differential drug sensitivity profiles between the two subtypes, as depicted in Figure 7A and Supplementary Figure S2A, which is in alignment with the prior prognostic findings. Noteworthy contrasts emerged: CRCS1 exhibited heightened sensitivity to certain agents, like gefitinib, erlotinib, dasatinib, saracatinib, and lisitinib, while CRCS2 showcased a stronger response towards temsirolimus, pazopanib, imatinib, crizotinib, and axitinib. Highlighted in Figure 7B,C are the top 10 candidate drugs showcasing the most significant variances between the 2 subgroups. In further exploration, CRCS1 surfaced as being particularly reactive to CGP.082996, A.443654, CMK, and assorted compounds, while CRCS2 exhibited higher sensitivity to CCT007093, PD.0332991, and VX.702, along with other pharmaceutical agents. This characterization underscores the potential for tailored therapeutic interventions dependent on the specific molecular signatures inherent to each subgroup.

### 3.6. Construction and Validation of a Gene Risk Model Related to Cancer Stemness

**Figure 8:**
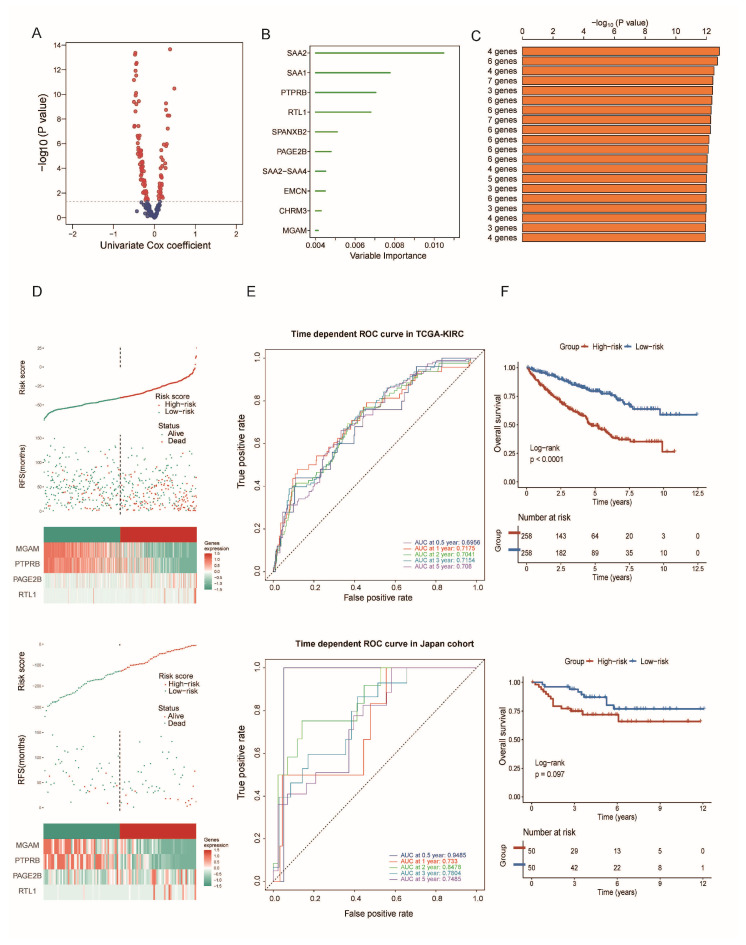
Construction and verification of a risk score system based on DEGs between CRCS1 and CRCS2. (**A**) Volcano plot indicating Cox coefficient and *p* value of each DEG. (**B**) Forest plot showing the prognostic importance of each DEG. (**C**) Combination of different risk score systems. (**D**) Risk score distribution of each sample in the TCGA-KIRC (up) and Japan-KIRC cohorts (down). (**E**) ROC curves indicate the performance of the risk score system based on MGAM, PTPRB, PAGE2B, and RTL1 in the TCGA-KIRC (up) and Japan-KIRC cohorts (down). (**F**) Kaplan–Meier curves show different clinical outcomes of patients with high or low risk scores in the TCGA-KIRC (up) and Japan-KIRC cohorts (down).

Having observed distinct clinical outcomes and biological complexities between the two subtypes, a risk model was precisely constructed by leveraging the subtype features. Initial stages involved a univariate Cox regression analysis to select signatures related to prognosis, as demonstrated in Figure 8A. Subsequent utilization of the random forest supervised classification algorithm effectively sieved through the data to identify a subset of 10 genes critical to the predictive model, as displayed in Figure 8B. We selected the most concise and effective prognostic model, which comprises four molecules: MGAM, PTPRB, PAGE2B, and RTL1. Based on the median risk score, patients were classified into high-risk and low-risk groups (Figure 8C,D). The analysis unveiled significantly divergent outcomes, accentuating the less favorable prognosis experienced by high-risk group individuals relative to their low-risk counterparts (Figure 8E,F).

### 3.7. Biological Role of SAA2 in ccRCC

Since we observed SAA2 to be highest in the prognostic ranking in the random forest, we explored the potential molecular biology role of this molecule in ccRCC. With the aim of unveiling the single-cell transcriptomic dynamics of SAA2 within cancer and its implications on cellular divergence, a meticulous examination of pan-cancer single-cell RNA sequencing databases was pursued (Figure S3A). Revelations pointed towards accentuated SAA2 expression within fibroblasts in KIRC (Figure S3B). In addition, distinct RCC single-cell RNA-seq datasets illuminated SAA2 visibility across various cancers, predominantly in fibroblasts, endothelial cells, and tumor cells. Spatial transcriptomic insights from RCC models accentuated endothelial cell prominence in SAA2 expression, which is intricately involved in orchestrating interactions among NK cells, plasma cells, neutrophils, and fibroblasts (Figure S3C,D). Survival analysis, in conjunction with heightened SAA2 expression, outlined reduced disease-specific survival, overall survival, and a progression-free interval, signaling a trend towards advanced tumor staging (Figure S3E,G). Through correlation analysis, we found that SAA2 might be involved in classic signals involving in cancer progression, including cytokine and receptor interaction, primary immunodeficiency, drug metabolism, IL6-JAK-STAT3, E2F, and the G2M checkpoint (Figure S4A,B). Furthermore, GSEA and KEGG outcomes further fortified associations between escalated SAA2 levels and complement and coagulation cascade activation (Figure S5A–C). Correlation analysis between SAA2 levels and distinct markers, such as immune stimulators, chemokines, immune inhibitors, and human leukocyte antigen, underscored positive associations with the immune checkpoint molecules CTLA4, LAG3, and PDCD1 (Figure S5D,E).

## 4. Discussion

ccRCC is recognized as a disease with substantial heterogeneity and immunogenic traits. The complexity of its heterogeneity and immunogenic nature contribute to the less than desirable outcomes seen with immunotherapy in ccRCC patients [38,39]. Consequently, there is a pressing need to delineate the distinct molecular subtypes of ccRCC, predict patient survival outcomes more accurately, and potentially augment the efficacy of immunotherapeutic strategies.

A multitude of research has identified substantial variability among cells within tumor masses, with a particular subset known as CSCs possessing the capability for ongoing self-renewal, bidirectional differentiation, and the performance of asymmetric cell division. Representing a smaller proportion within the larger cellular ensemble, CSCs have a critical role in tumor genesis and are deeply linked to the recurrence of cancer, its spread, and its resistance to chemotherapy. The question of CSC origins remains debated, with the hierarchical and stochastic models being central theories. Baumann and colleagues have defended the classical hierarchical model, suggesting that self-renewal and division are restricted to a select group of cancer cells [40,41,42]. Conversely, increasing research [43,44,45,46] champions the stochastic model, positing that given the right signals, any cancer cell might be reprogrammed into a CSC. Furthermore, Wang and colleagues have pointed out that the two-way switch, also known as plasticity within cancer cells, could pose a significant hurdle in treating cancer [47,48]. Thus, delineating the intricacies of cancer cell plasticity and pinpointing its governors could mitigate the risk of cancer cells morphing into CSCs, potentially lowering the recurrence rate. Despite growing evidence underscoring the critical role of CSCs in cancer’s tenacity and resistance to therapy, there is a pressing need to unearth the fundamental processes responsible for sustaining tumor stemness and targets related to stemness that have potential clinical application.

In our research, we dissected stemness genes within tumors across TCGA-KIRC cohort by delving into multi-omics databases. We adopted k-means from R package ConsensusClusterPlus to perform consensus clustering analysis, and patients with ccRCC were segmented into CRCS1 and CRCS2 based on their tumor stemness score matrix. It should be noted that the consensus clustering strategy has been widely applied in cancer research, since it is straightforward and easy to interpret, has a fast computation speed, and is well-suited for large-scale datasets when comparing to affinity propagation, agglomerative clustering, mean shift clustering, bisecting k-means, DBSCAN, OPTICS, and BIRCH [49]. For example, Li et al. applied multi-omics datasets including proteomic and phosphoproteomic profiling of colon cancer based on unsupervised clustering, which could successfully distinguish case with high risk of metastasis events [50]. Luo and colleagues adopted circulating tumor DNA methylation profiles and performed an unsupervised clustering based on risk related signatures, which could stratify high risk group among patients with colorectal cancer [51]. Recently, Hu et al. integrated multi-omics including genomic, transcriptomic, proteomic, metabolomic, and spatial transcriptomes and metabolomics across 100 patients with ccRCC and identify a subgroup with a poor clinical outcome and characterized them with de-clear cell differentiation based on unsupervised clustering [52]. All of the abovementioned results indicated that consensus clustering analysis was a promising approach to decode tumor heterogeneity, especially under the multi-omics level. In detail, CRCS1, characterized by a worse prognosis in this work, was associated with advanced pathological grades, elevated tumor mutation loads, a heightened metabolic activity and a predisposition towards an immunosuppressive environment. Additionally, a prognostic model tailored to these subgroup traits exhibited promising outcomes in both the initial and subsequent patient cohorts. Our investigation further indicated that an extraordinary level of tumor stemness could portend a worsen prognosis.

Combining immune checkpoint inhibitors with tyrosine kinase inhibitors is the preferred frontline treatment for ccRCC [53]. Still, not all patients experience favorable outcomes from immunotherapy; objective response rates to these drug combinations can vary widely, from approximately 41% to 71% [54,55,56,57,58]. A potential strategy to overcome this issue may include combining these treatments, potentially in coordination with assessments of tumor stemness, to complement immunotherapy. It is a recognized phenomenon that CSCs generally exhibit resistance to chemotherapy and radiotherapy, primarily because CSCs often remain in a dormant state [59,60,61]. CSCs from various cancer types, akin to normal stem cells, tend to proliferate at a reduced pace and are preserved in the G0 phase of the cell cycle through sophisticated regulatory controls [62]. Research by Liu et al. revealed that breast cancer CSCs can alternate between mesenchymal-like and epithelial-like states, influencing the tumor’s propensity to grow, spread, and establish itself at different locations [63]. Gan and collaborators found that EZH2 is capable of initiating an epithelial–mesenchymal transition in gastric cancer cells by influencing the Akt/PTEN axis [64]. Meanwhile, Musella et al. discovered that Type I IFNs can initiate a temporary, protective anticancer reaction, via KDM1B, which can restrain the expansion of CSCs and improve long-term treatment outcomes. Takeishi and colleagues explored dual strategies for targeting CSCs by either encouraging or impeding their progression through the cell cycle [65]. Overall, the extensive range of studies suggests that cancer cells’ capacity to shift between stem-like and differentiated states allows adaptation to varying stimuli within the microenvironment. This corroborates findings related to the CRCS2, stemness relative depressed group, in our study, which demonstrates a reduced pathological grade, a diminished presence of immune factors, and a lower immune cell presence, and which is characterized by irregular metabolic processes involving fatty and bile acids.

The CSC model has piqued considerable interest, seeing as it encapsulates cellular diversity within tumors—incorporating genetic, epigenetic, and TME influences—as a way to elucidate the persistence of tumors and their relapse after treatment, in addition to pinpointing new avenues for creating anticancer therapies. Current strategies principally focus on eradicating CSCs by targeting characteristics they share with normal stem cells. However, the narrow therapeutic window presents a significant challenge for these methods. Employing combination therapies, which are informed by a deeper grasp of CSC-specific markers, tweaking drug dosages aligned with their biological roles, and crafting thoughtful strategies using data from suitable preclinical studies, could enhance the therapeutic window and specificity of these treatments. Moreover, cutting-edge pharmaceuticals aim at disrupting the developmental signal transduction routes that maintain the CSCs’ survival and renewal, chiefly targeting the Hedgehog (Shh), Notch, and Wnt pathways. It has been shown that blocking the Shh pathway can eliminate imatinib-sensitive or -resistant BCR-ABL+ cells [66]. Wnt signaling, crucial for regulating the well-acknowledged stem cell marker CD44, has been observed to interact directly with this protein’s promoter to create a reservoir for resistance [67]. Research suggests a bidirectional interaction between CD44 and the Wnt pathway; CD44 latches onto β-catenin, activating Wnt and leading to enhanced resistance to cisplatin [68]. The binding of Hedgehog ligands, including Indian Hedgehog (IHH), Sonic Hedgehog (SHH), and Desert Hedgehog (DHH), to their receptor Patched (PTCH) reduces the repression of Smoothened (SMO). This results in the activation of SMO, which then triggers the glioma-associated oncogene family (GLI) signaling sequence, acting as a precursor to the transcription of genes underpinning the Hedgehog pathway [69,70,71]. Sonidegib (LDE225), an SMO antagonist, has earned FDA clearance [72,73], championed by its notable efficacy in treating advanced and metastatic basal cell carcinoma, as demonstrated by Dummer et al. Vismodegib (GDC-0449), another antagonist of SMO, also delivered promising outcomes in metastatic basal cell carcinoma and received FDA approval [74,75]. Nonetheless, findings from Berlin, Catenacci et al. indicated that vismodegib failed to magnify the impact of standard chemotherapy for patients with metastasized colon and pancreatic cancers [76,77]. Our research posits that CRCS1, a hypoxic variant of ccRCC demonstrated by the notable elevation of transcription factors that govern low-oxygen-adaptive genes, could see enhanced therapeutic impacts when immune checkpoints and signal transduction pathways are simultaneously inhibited. This multipronged approach has the potential to amplify genomic instability within the tumor and reinstate immune equilibrium, bolstering the effectiveness of drugs.

Beyond tumor stemness, genes connected to the cell cycle hold significant influence over the conservation of genomic stability, the evolution of cancer, and resistance to treatments [78]. Through analyses using KEGG and GO, it was noted that tumor stemness intersects with the dysregulation of several cancer-related signaling pathways, including those governing lipid metabolism and the cell cycle. CRCS1 displays a state of hypoxia, with some pathways, like bile acid metabolism and fatty acid metabolism, being considerably more active in this subtype. Tumor stemness has also been linked with genes that manage the cell cycle, such as those responsible for chromosome segregation. The cell cycle functions to prevent the acquisition of malignancy in three primary ways: (i) triggering disorderly homologous recombination during the G1 phase in cancer cells; (ii) provoking mitotic mutations within cancer cells; or (iii) eliminating checkpoints within the unidirectional cycle [79]. Significant to the development of renal cancer is the role that cell cycling plays. Liu et al. demonstrated that cyclovirobuxine significantly reduces the expression of Snail—an EMT-associated transcription factor—thereby causing a block in the S phase and promoting apoptosis through the suppression of the AKT, STAT3, and MAPK signaling pathways [80]. Pahwa and colleagues discovered that SNX2112 induces cell cycle arrest in the G2/M phase and prompts apoptosis, consequently abating the proliferation of kidney cancer cells [81]. Notably, the p53 molecular mechanism maintains transcriptional activation of genes pivotal for ceasing the cell cycle and orchestrating responses to chemotherapeutic drugs. Importantly, pathways involved in DNA repair were also notably active among CRCS1. These findings suggest that targeting DNA damage repair mechanisms may represent a potential therapeutic approach for patients within the CRCS1 subtype. Specifically, drugs, such as PARP inhibitors or PARPi, and other agents targeting DNA damage response (DDR) pathways could be promising treatment strategies for patients with elevated stemness scores in the CRCS1. Previous work from Ji and colleagues suggested that treatment with PARPi could enhance therapy efficacy of CAR-T cell therapy through activating the cGAS-STING signal [82]; Zhou et al. found that a combination of PARPi and DNA hypomethylation agents could effectively inhibit tumor progression among SETD2-deficient ccRCC [83].

Importantly, we identified that SAA2 could be treated as a novel prognostic and therapeutic target for ccRCC. Previous works also highlighted the role of SAA2 in tumor progression [84]. For instance, Stone et al. reported that SAA2 derived from hepatocytes interacts with TLR2, which inhibits dendritic cells (DCs) and anti-tumor T cell immunity, thereby preventing the immune surveillance of tumor cells [85]. Additionally, research by Wu’s team indicated that CXCL6, produced by highly invasive tumor cells, stimulates the release of SAA from damaged hepatocytes, encouraging macrophage infiltration and polarization via FPR1 and TLR2 receptors [86]. Zila et al. observed across multiple datasets that overexpression of SAA family members, including SAA1 and SAA2, predicts lower response rates to immune checkpoint inhibitors (ICIs) in metastatic melanoma patients [87]. Furthermore, Wang and colleagues noted that the aberrant expression and release of SAA2 regulated by WDR4/PML promotes lung cancer progression by increasing intertumoral Tregs and M2-like macrophages while reducing CD8+ T cell infiltration [88]. Lastly, Cooley et al. identified SAA2 as a promising soluble prognostic and predictive biomarker for RCC through serially passaging mouse models and large-scale multi-omics datasets [89]. Collectively, these studies suggest that SAA2 is not only a pro-cancer molecule, particularly in RCC, but also facilitates tumor progression through various mechanisms, including inducing immune suppression or evasion. Moreover, we noticed that the hypoxia signal was activated in CRCS1, which might contribute to immune evasion within this subtype. Previous studies found that hypoxia in ccRCC activates various signaling pathways that lead to immune suppression. One of the key mediators of this process is hypoxia-inducible factor 1-alpha, which is stabilized under low-oxygen conditions. HIF-1α induces the expression of several immune checkpoint molecules, including PD-L1 and CTLA-4, on tumor cells and immune cells, respectively, thereby inhibiting T cell activation and promoting an immunosuppressive environment [90]. Moreover, hypoxia alters the recruitment and function of immune cells within the TME. It has been shown that hypoxic conditions promote the accumulation of myeloid-derived suppressor cells (MDSCs) and regulatory T cells (Tregs), both of which are critical for maintaining an immunosuppressive milieu. MDSCs are known to inhibit T cell responses through various mechanisms, including the production of reactive oxygen species (ROS) and arginase, which deplete essential amino acids from the TME [91]. Tregs, on the other hand, are expanded in response to hypoxic signals and can actively suppress effector T cell functions, further dampening anti-tumor immunity. Interestingly, we found that hypoxia was activated in CRCS1, and the immune checkpoint-related signature, Treg, M2 macrophage were also enriched, which suggested that targeted hypoxia in such a group could reactivate tumor immunity in such a subtype.

ccRCC exhibits a notably high TMB, and the genes that regulate tumor stemness are connected to genomic mutations, affecting biological functions. Mutation rates were found to be elevated in the CRCS2 subtype compared to CRCS1, with CRCS2 harboring a cohort of frequently mutated genes, such as BAP1, mTOR, and KDM5C. Extensive research has established BAP1 as a critical tumor suppressor gene implicated in ccRCC. BAP1 participates in various cellular processes, including DNA repair and gene transcription within the nucleus, and carries out roles related to cell death regulation and mitochondrial metabolism in the cytoplasm. In the context of renal cell carcinoma, BAP1 mutations correlate with cell proliferation, metastatic potential, high tumor grades, and unfavorable prognoses [92,93,94]. The mTOR gene is another classic mutation target seen in kidney cancer and is a key focal point for emerging treatment strategies in ccRCC, combining mTOR pathway inhibitors with an immune checkpoint blockade. Drugs targeting mTOR pathways, such as lenvatinib, everolimus, and sirolimus, have gained regulatory approval for treating kidney cancer [95,96,97]. Regarding KDM5C, a gene encoding a histone demethylase, its mutations influence a wide array of biological processes. According to Federico et al., ccRCC patients harboring KDM5C mutations demonstrated a significant increase in visceral adipose tissue relative to those carrying VHL mutations [98,99,100]. Moreover, research by Zheng and colleagues suggests that mutations in KDM5C can stimulate ccRCC cell proliferation through restructuring glycogen metabolism and constraining ferroptosis [99]. This evidence underscores the complex interplay between genetic alterations, tumor biology, and susceptibility to therapy in ccRCC.

This investigation has highlighted that the NRF2 and PI3K signaling pathways are substantially affected in the CRCS2 subtype. These pathways are well-documented for their critical roles in kidney cancer progression, recognized as hallmark oncogenic mutation pathways. In CRCS2, we observed an escalation in copy number variations. Research by Fernandes et al. has emphasized notable copy number changes in ccRCC, including increases in regions 5q, 7p, and 16q, and decreases in 3p, 14q, and 6q [100], a pattern that aligns with our findings. Consequently, it appears that genes associated with tumor stemness contribute to tumor heterogeneity by engaging with genomic mutations.

For the analysis of drug sensitivity, we extracted drug response metrics, namely IC50 values, from the GDSC database. The investigation revealed that the CRCS2 group generally exhibited a less favorable response to most drugs, echoing prior prognostic data. CRCS1 was more responsive to certain drugs, such as gefitinib and lisitinib, whereas CRCS2 demonstrated higher sensitivity towards pazopanib and imatinib, with the latter showcasing enhanced effects on CRCS1. We then identified a set of 138 small molecule compounds as potential ccRCC treatment options. As illustrated in Figure 5C,D, the top 10 drugs demonstrating the largest differential in efficacy between the subtypes were isolated. Specifically, CRCS1 showed sensitivity to certain compounds, like CGP.082996, A.443654, and CMK; conversely, CRCS2 was more susceptible to treatment with CCT007093, PD.0332991, and VX.702. As previously highlighted, genes connected to tumor stemness influence the potency of cancer treatments. Subtypes within ccRCC exhibit varied drug sensitivities, which could inform clinical decision-making. In our study, we pinpointed several inhibitors that hold promise for the CRCS2 subtype: CCT007093, a PPM1D inhibitor; palbociclib, a highly selective CDK4/6 inhibitor; and VX-702, a p38 MAPK inhibitor, all showing greater effectiveness in targeting CRCS2.

In general, based on stemness-related signatures, our study constructed and verified two novel subtypes of ccRCC which stratify patients’ prognosis and aid in choosing the correct treatment. Nonetheless, our research into the regulatory landscape of tumor stemness within ccRCC is not without its caveats. Some conclusions are derived from extensive bioinformatic analyses and warrant subsequent corroboration through laboratory experimentation, particularly concerning the specific roles of tumor stemness regulators. Furthermore, we utilized clustering algorithms from ConseususClusterPlus R package, which has been widely applied across pan-cancer clustering works. Notably, this algorithm can struggle with non-spherical clusters and is sensitive to the initial selection of cluster centers, which necessitates a predefined number of clusters [101]. Therefore, we hope that more advanced algorithms can overcome the limitations of the data and maximize the retention of heterogeneity between samples for molecular typing studies of larger cancer populations. Additionally, the predictive model could be skewed by confounding variables, such as patient demographics and geographic factors, necessitating further validation using additional, varied datasets to solidify our risk model’s credibility. However, we noticed that SAA2 could be treated as a potential biomarker and as a therapeutic target for advanced ccRCC, and the role of SAA2 was investigated through correlation analysis. We admit that additional in vivo and vitro experiments are needed to better understand the biological function of SAA2.

## 5. Conclusions

To summarize, this study delineates two molecular subtypes of ccRCC, utilizing CSCs as a biomarker and delving into the role of regulatory genes that drive stemness within kidney cancer. We suggest that strategies aimed at targeting genes related to tumor stemness may provide a novel avenue to counteract drug resistance in ccRCC. By altering the tumor microenvironment and thwarting the cancer cells’ immune evasion tactics, such therapeutic measures have the potential to amplify the success of existing immunotherapy treatments. The innovation of new drugs that impede pathways critical to the sustenance and proliferation of CSCs might also show a complementary effect when used alongside immunotherapies, paving the way for more comprehensive tumor elimination strategies. Ultimately, our research enhances the comprehension of the dynamic relationship between CSCs and ccRCC and delineates pivotal directions for the conception of precision medicine approaches that can improve survival and quality of life for patients affected by ccRCC.

## Figures and Tables

**Table 1:** Baseline characteristics between patients of the CRCS1 and CRCS2 subgroups.

	CRCS1	CRCS2	*p*.Overall
	*N =* 224	*N =* 282	
T:			0.001
T1	98 (43.8%)	162 (57.4%)	
T2	28 (12.5%)	39 (13.8%)	
T3	89 (39.7%)	79 (28.0%)	
T4	9 (4.02%)	2 (0.71%)	
N:			0.112
N1	10 (8.26%)	5 (3.18%)	
NX	111 (91.7%)	152 (96.8%)	
M:			0.560
M1	41 (71.9%)	34 (79.1%)	
MX	16 (28.1%)	9 (20.9%)	
grade:			0.001
G1	5 (2.23%)	7 (2.48%)	
G2	81 (36.2%)	136 (48.2%)	
G3	89 (39.7%)	112 (39.7%)	
G4	46 (20.5%)	25 (8.87%)	
GX	3 (1.34%)	2 (0.71%)	
stage:			0.013
i	96 (42.9%)	158 (56.0%)	
ii	24 (10.7%)	31 (11.0%)	
iii	59 (26.3%)	58 (20.6%)	
iv	45 (20.1%)	35 (12.4%)	
sex:			0.061
female	87 (38.8%)	86 (30.5%)	
male	137 (61.2%)	196 (69.5%)	
age	60.2 (12.0)	60.5 (12.4)	0.769
OS:			<0.001
0	127 (56.7%)	210 (74.5%)	
1	97 (43.3%)	72 (25.5%)	
OS.time	1210 (879)	1560 (1018)	<0.001
PFI:			<0.001
0	134 (59.8%)	215 (76.2%)	
1	90 (40.2%)	67 (23.8%)	
PFI.time	1004 (850)	1373 (980)	<0.001

## Data Availability

The original contributions presented in the study are summarized in Section 2, and further questions can be addressed to the corresponding authors.

## Supplementary Materials

The following supporting information can be downloaded at: https://www.mdpi.com/article/10.3390/biomedicines12102171/s1, Figure S1. Functional enrichment analysis. (A,B) Gene ontology (GO) analysis was applied to the differentially expressed genes (DEGs) between CRCS1 and CRCS2. (C) A heatmap illustrating the expression levels of TME between CRCS1 and CRCS2. Figure S2. (A) The IC50 values of the targeted molecular drugs were compared between the 2 subtypes. (B) A survival analysis was performed for the overall survival of the 2 subtypes in the IMvigor210 (left), GSE22541 (middle), and Japanese cohort (right). Figure S3. (A) Expression profile of SAA2 across publicly available pan-cancer scRNA-seq databases. (B) Expression profile of SAA2 in RCC-related scRNA-seq databases. (C) Spatial slice and Seurat clusters colored by cell types. (D) Heatmap showing Pearson’s correlation between cell abundance in RCC. Cell abundance was represented by ssGSEA score calculated using marker genes from snRNA-seq. (E) Meta-analysis demonstrating the impact of SAA2 on the disease-free interval (DFI), disease-specific survival (DSS), overall survival (OS), and progression-free interval (PFI). (F) Expressions of SAA2 in each tumor stage in TCGA-KIRC. (G) The Kaplan–Meier curves for PFI (left), DSS (middle), and OS (right) in the two risk signature groups within the TCGA-ccRCC cohort are displayed. Figure S4. The results of gene sets enrichment analysis of (A)KEGG and (B) GSEA in ccRCC based on SAA2 expression. Figure S5. (A) KEGG hierarchy-based functional enrichment analysis for SAA2, illustrating the biological processes and pathways associated with SAA2. (B) GSVA based on the expression of SAA2 was conducted to evaluate gene set activity and its variation. (C) GSVA using the Fgsea package was performed based on SAA2 expression to explore its impact on the gene expression landscape. (D) A multi-layered heatmap based on SAA2 expression, highlighting the immunostimulator, chemokines, immunoinhibitor, and human leukocytoantigen patterns of gene expression and differences. (E) Fisher’s exact test and scatter diagrams for immune checkpoint markers.
