# Supplementary figures and images for: A Multi-Omics Prognostic Model Capturing Tumor Stemness and the Immune Microenvironment in Clear Cell Renal Cell Carcinoma
Source: Biomedicines. 2024 Sep 24;12(10):2171. doi: 10.3390/biomedicines12102171 (PMC11504857; doi:10.3390/biomedicines12102171)

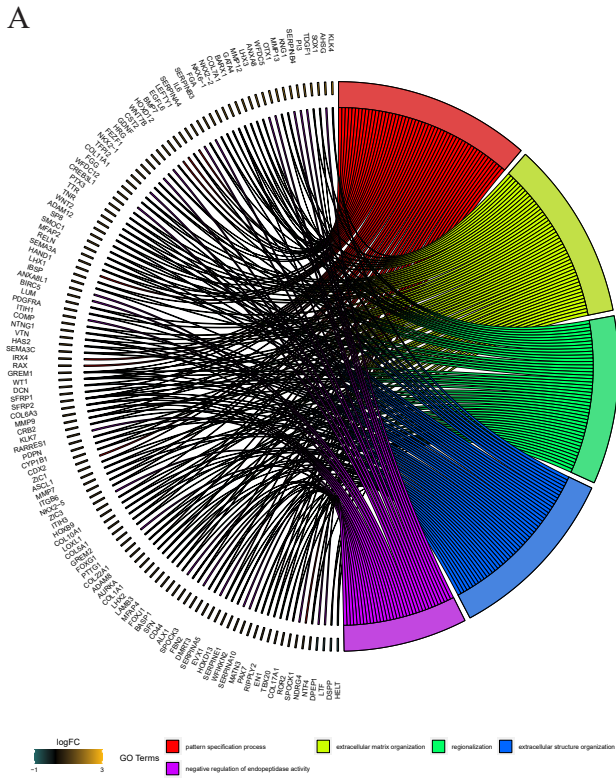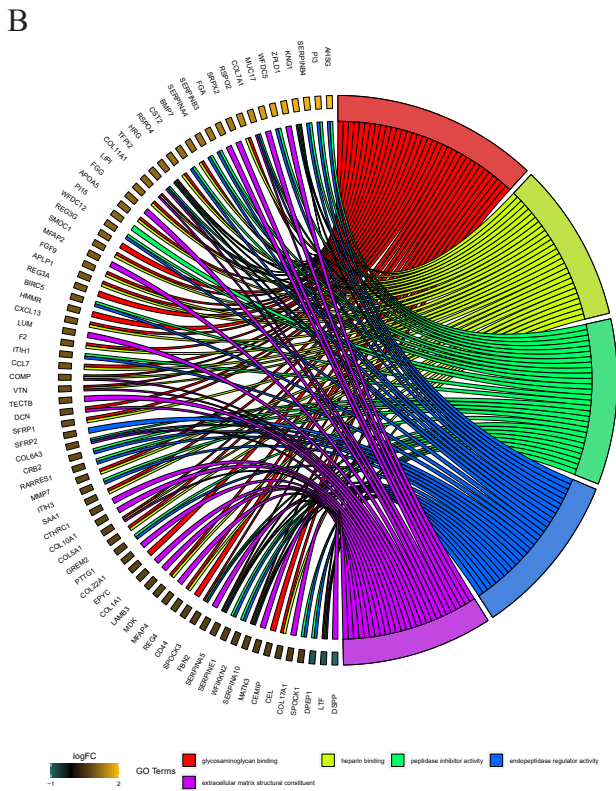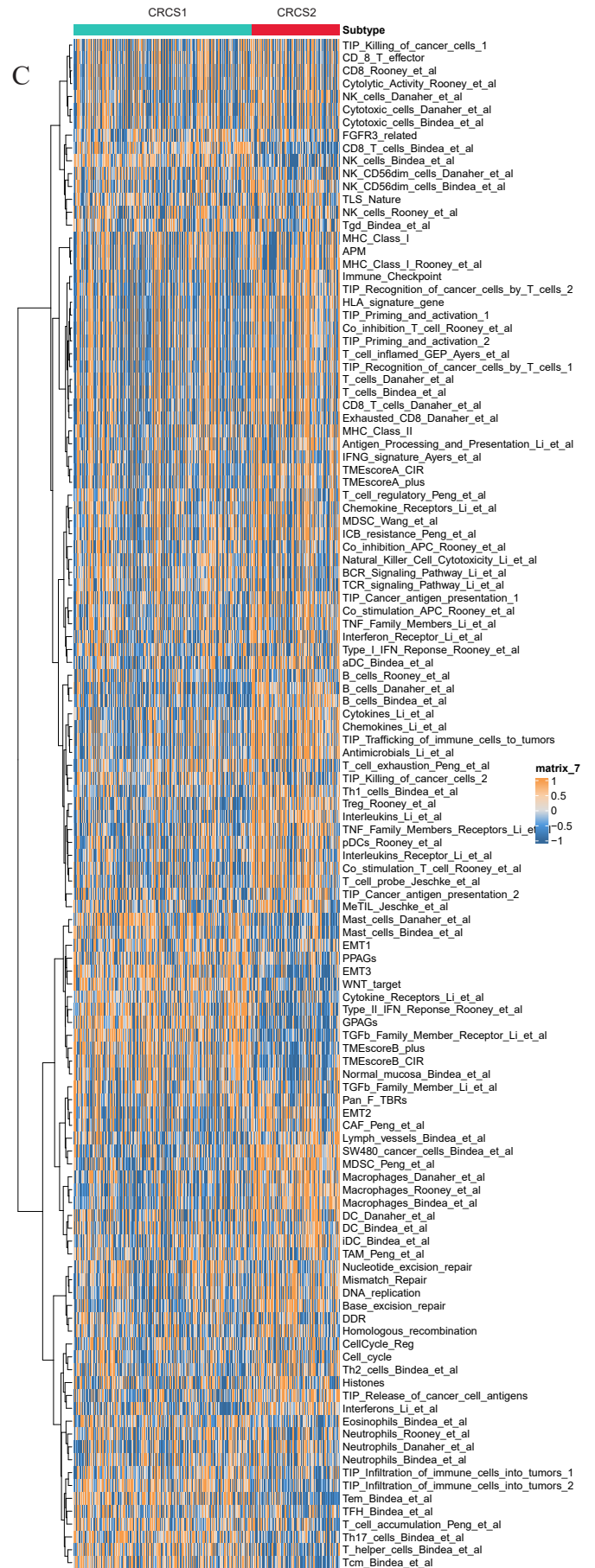

Supplement: Supplementary file 1 [file biomedicines-12-02171-s001.zip › FigureS1.pdf]

A

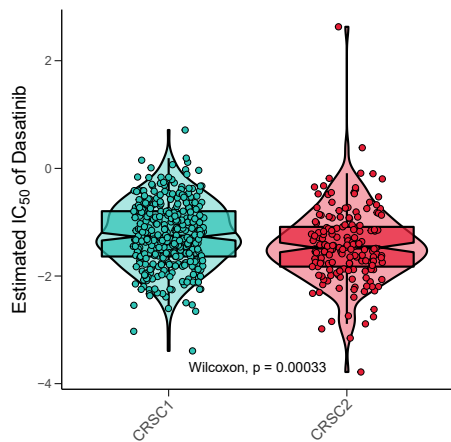

B

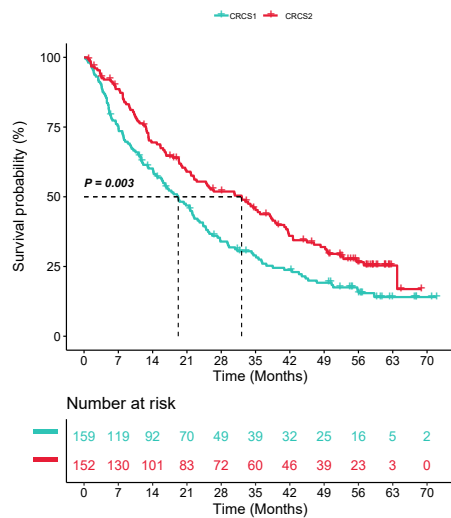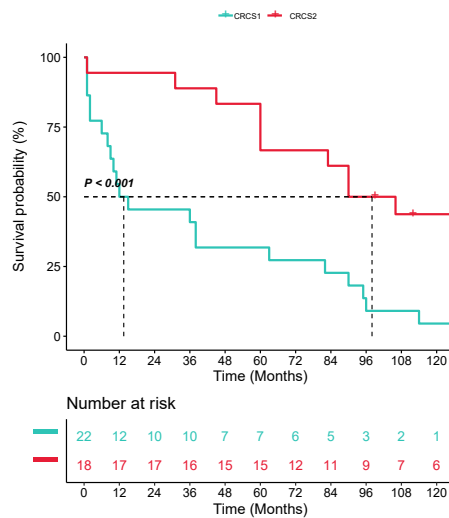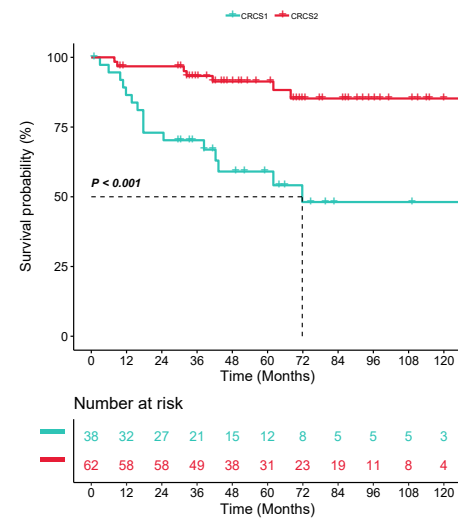

Supplement: Supplementary file 1 [file biomedicines-12-02171-s001.zip › FigureS2.pdf]

A

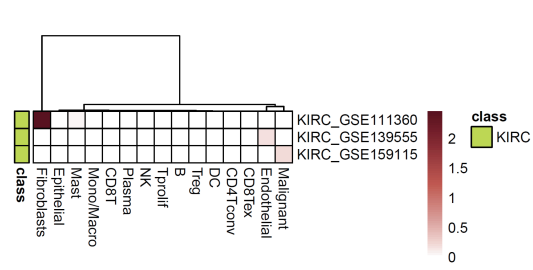

B

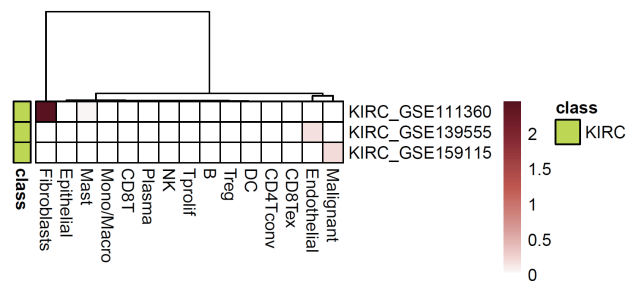

C

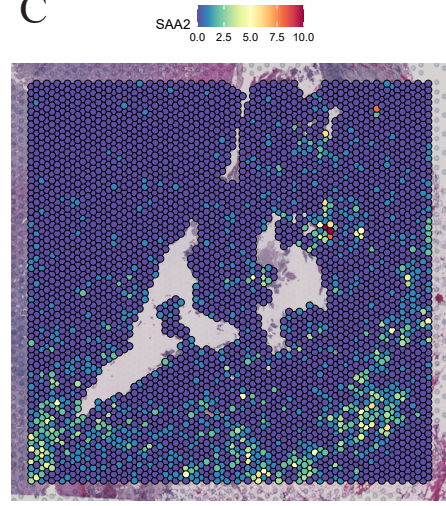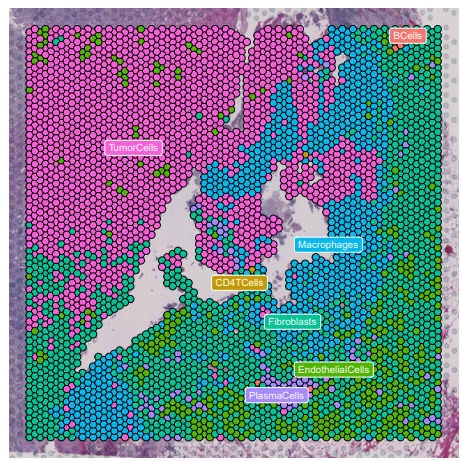

D

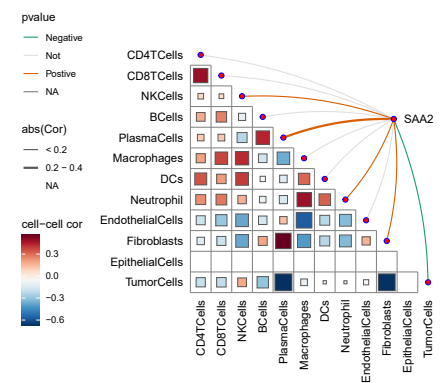

E

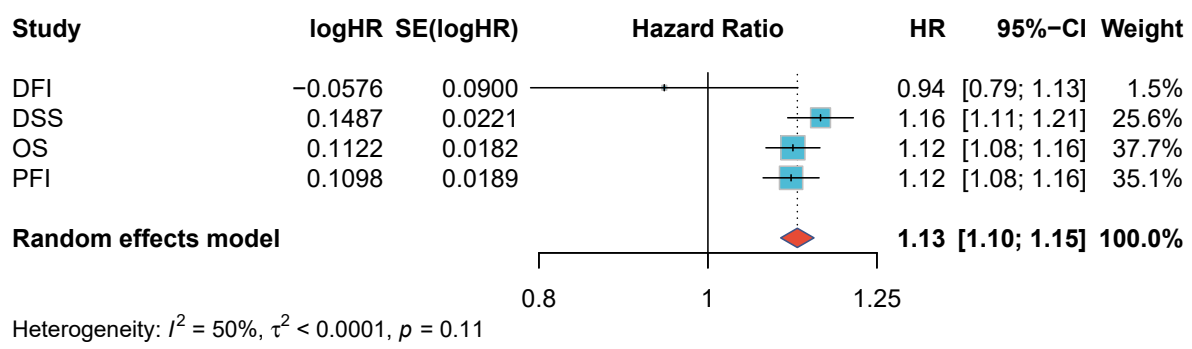

F

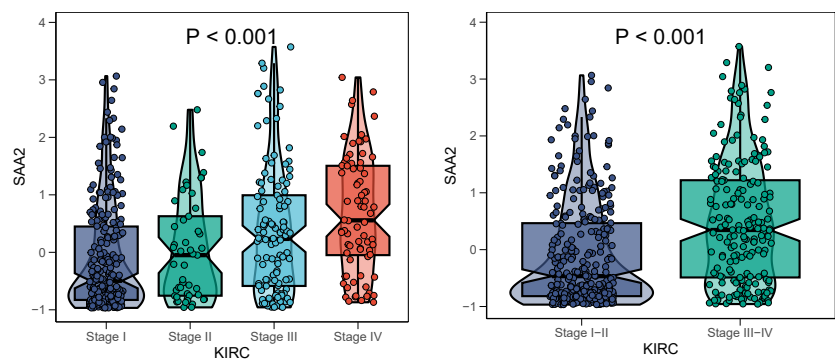

G

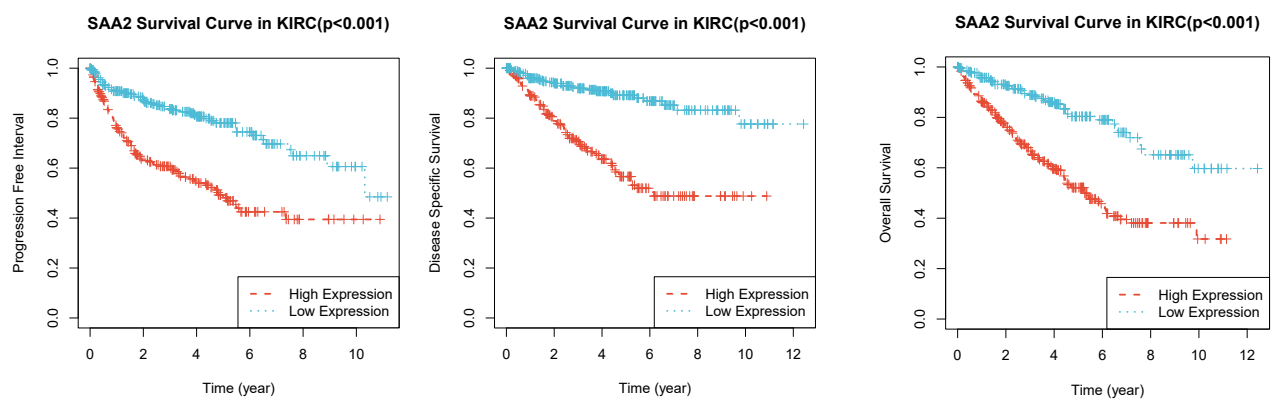

Supplement: Supplementary file 1 [file biomedicines-12-02171-s001.zip › FigureS3.pdf]

A

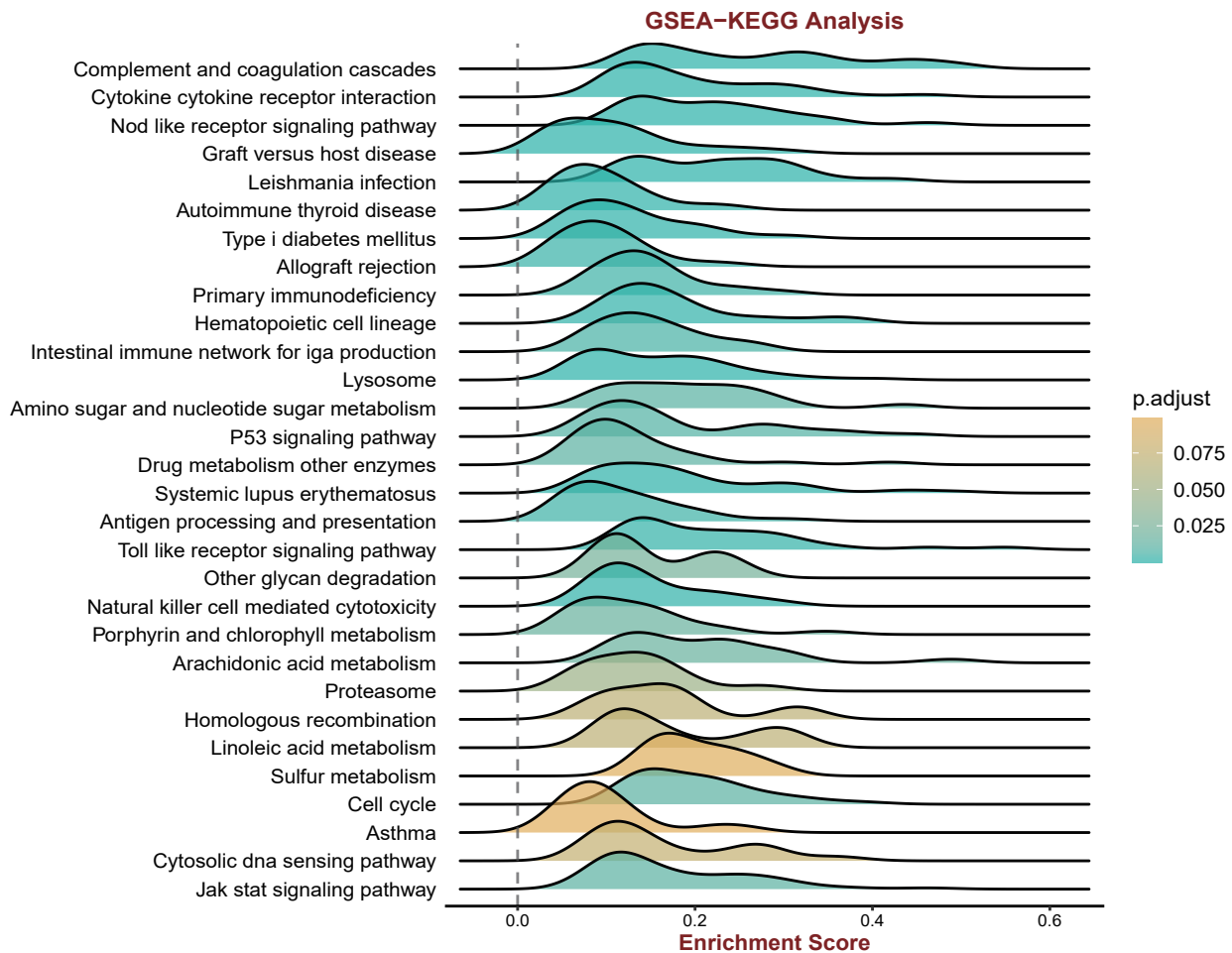

B

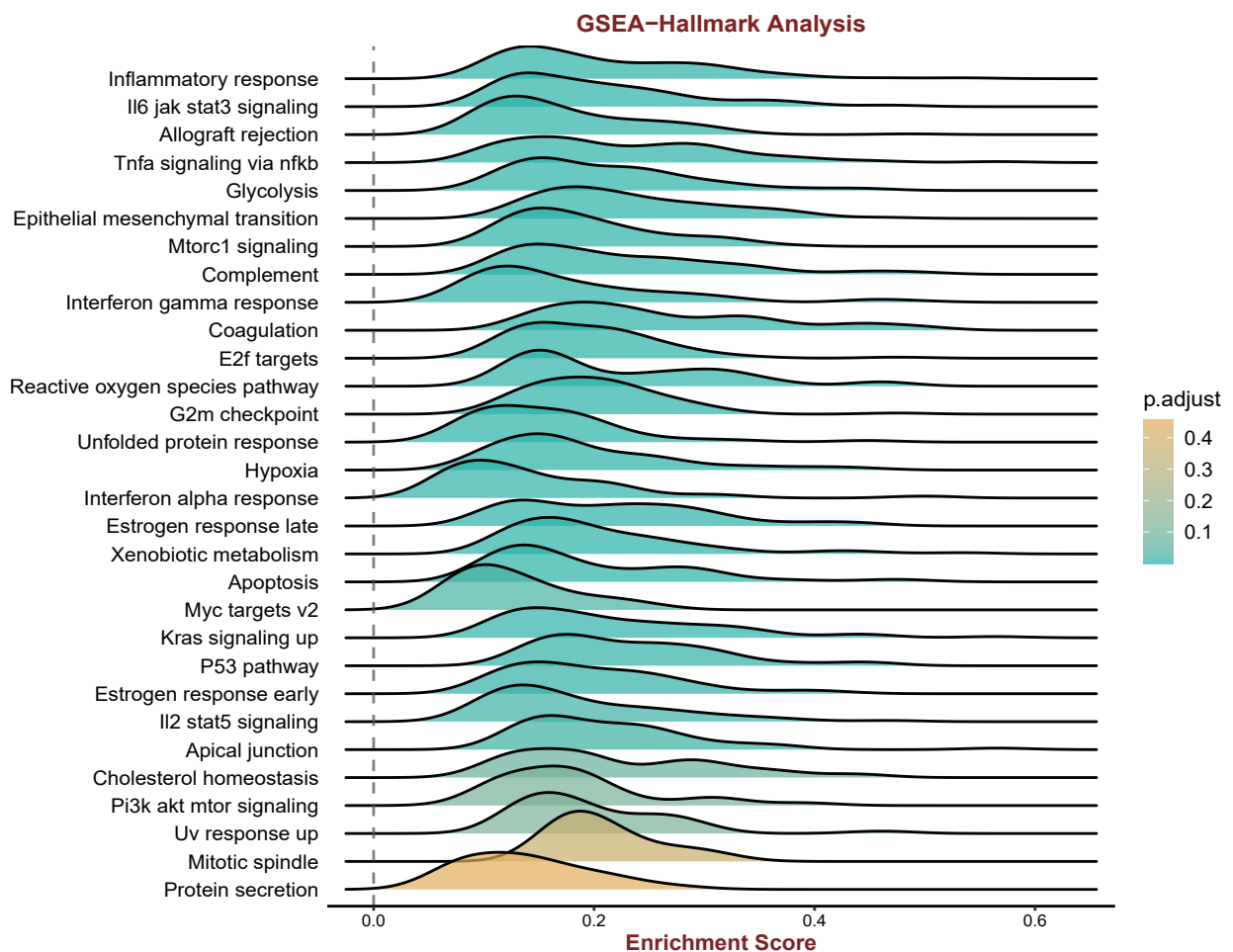

Supplement: Supplementary file 1 [file biomedicines-12-02171-s001.zip › FigureS4.pdf]

A

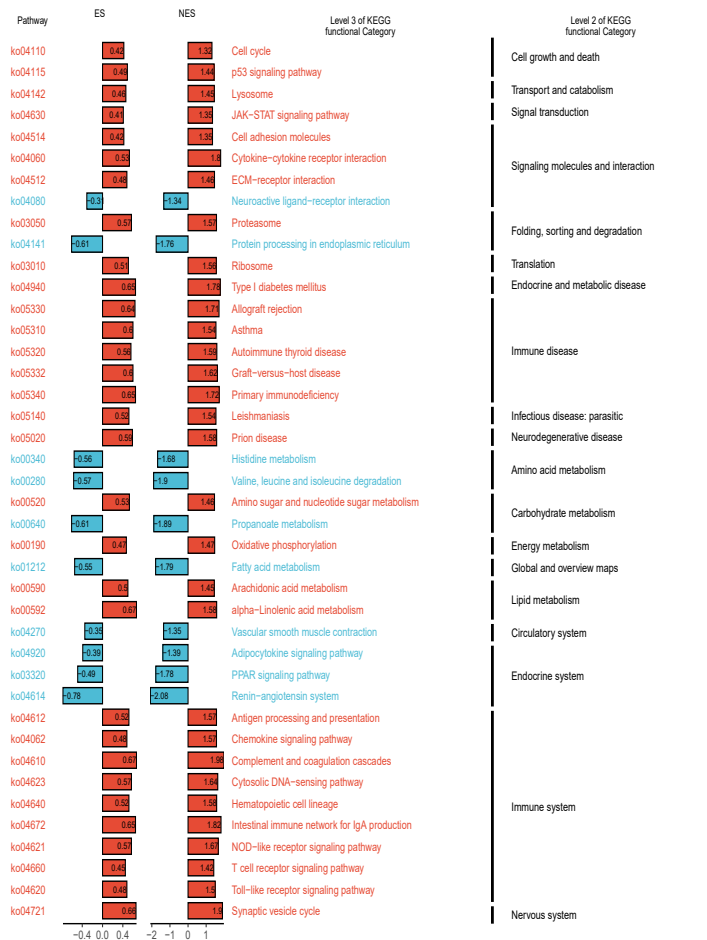

B

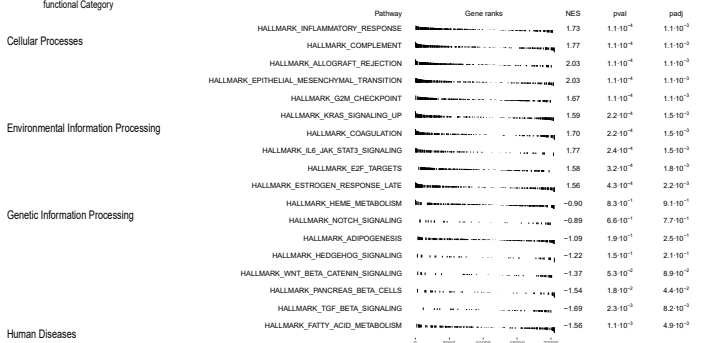

C

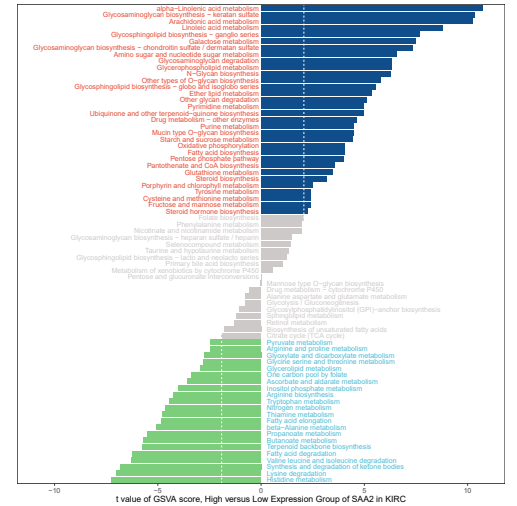

Supplement: Supplementary file 1 [file biomedicines-12-02171-s001.zip › FigureS5.pdf]
